# Curcumin Aattenuates the Expression of Metalloprotease (AHA_0978) and Serine Protease (AHA_3857) Genes in Aeromonas Hydrophila

**DOI:** 10.31661/gmj.v12i.3038

**Published:** 2023-12-17

**Authors:** Mahdieh SobhZahedi, Hojjatollah Zamani, Mohammad Hossein YektaKooshali

**Affiliations:** ^1^ Department of Biology, Faculty of Science, University of Guilan, Rasht, Iran; ^2^ Razi Hospital, School of Medicine, Guilan University of Medical Sciences, Rasht, Iran

**Keywords:** Curcuma, Quorum Sensing, Aeromonas Hydrophila, Metalloprotease, Serine Protease

## Abstract

Background: Aeromonas hydrophila is a pathogenic bacterium responsible for various infections in humans and animals. Bacterial exoproteases are considered an important determinant in the pathogenicity of A. hydrophila. Serine protease and metalloprotease, that are regulated by the bacterial Quorum sensing (QS) system are important virulent factors in the pathogenicity of A. hydrophila. Anti-QS potential of curcumin has been reported, previously. In this work, we characterized the effect of curcumin on the expression of the metalloprotease and serine protease genes in A. hydrophila. Materials and Methods: The minimum inhibitory concentration (MIC) of curcumin was measured by the agar macro-dilution method and a sub-inhibitory concentration (1/2 MIC) was used in subsequent experiments. The expression level of the metalloprotease and serine protease genes among the treated and control bacteria was evaluated using quantitative PCR (qPCR) assay. Bacterial proteolytic activity was also measured by skim milk agar plate assay. Results: MIC of curcumin for bacterial strain was 1024 μg/ml curcumin, and at 512 µg/mL (1/2 MIC) it remarkably attenuated the expression of the metalloprotease and serine protease genes up to 66 and 77%, respectively. Also, the proteolytic activity of A. hydrophila was considerably reduced by curcumin. Conclusion: Due to the promising inhibitory effect on bacterial proteolysis, curcumin could be considered an anti-virulence agent against A. hydrophila.

## Introduction

Aeromonas hydrophila is an environmental bacterium that can be found in aquatic habitats (fresh or brackish water), and also in food products. It is resistant to a variety of antibiotics, able to grow in cold temperatures [1], and causes various diseases in cold and warm-blooded organisms. This bacterium is pathogenic for fish species, amphibians, and also humans. It can cause gastroenteritis (mostly in young children and people with immunocompromised systems or growth problems), cellulitis, myonecrosis and eczema (mostly in compromised or suppressed by medication immune systems), necrotizing fasciitis, skin infections, peritoneal peritonitis, bacteremia, meningitis, hemolytic uremic syndrome (kidney failure), arthritis, suspected infection, septicemia and urinary and genital tract infection in human [2][3][4]. Several virulent factors, including bacterial glycocalyx, toxins, and extracellular enzymes are associated with the pathogenicity of A. hydrophila. Bacterial proteases have a critical role in infection development and disease progression. The majority of virulent factors are regulated by the A.

hydrophila Quorum sensing (QS) system that is a intercellular communication via the release of autoinducer molecules that enable a bacterium to ‘‘sense’’ its population. Several functions such as conjugation, biofilm formation, and secretion of virulence factors are regulated by bacterial QS systems [5][6].

The importance of proteolytic activity in the pathogenesis of Aeromonas spp. has been demonstrated. The activity of proteases starts from the beginning of the growth phase and reaches its peak near the beginning of the stationary phase [7]. Serine protease and metalloprotease are very effective extracellular factors in the pathogenicity of A. hydrophila. These two factors are involved in attacking the host tissues, where the bacterium adheres to the host cell and proteases are secreted into the space between the cells and digest amino acids and oligopeptides. Damage to the membrane of host cells can cause widespread tissue damage and create a focus of infection by overcoming initial host defense. On the other hand, it can cause the activation of aerolysin and Glycine C-acetyltransferase (GCAT), which are two other important factors in the pathogenicity of this bacterium [8][9]. In A. hydrophila the expression of Serine protease and metalloprotease genes is regulated by the bacterial QS systems [5]. Curcumin (1,7-bis(4-hydroxy-3-methoxyphenyl)-1,6-heptadiene-3,5-dione), is a polyphenol compound that is mainly found in the rhizome of Curcuma longa (turmeric). Curcuma longa has been used in Asia as a medical herb, traditionally because of its anti-inflammatory, metabolic syndrome, anti-mutagenic, antioxidant, antimicrobial, rheumatoid arthritis (RA), and anticancer properties [10]. Quorum sensing inhibitory potential of curcumin has been reported, previously [11]. Tanhay et al. reported that biofilm formation in A. hydrophila is significantly inhibited by curcumin [12]. They also reported that the expression of ahyIR, the major QS system of A. hydrophila, was significantly inhibited by curcumin. Due to quorum sensing inhibitory activity of curcumin, the present work was performed to characterize the effect of curcumin on the expression of metalloprotease and serine protease genes in A. hydrophila ATCC 7966.

## Materials and Methods

**Table:** Table1. The sequence of the AHA_3857 and AHA_0978 primers

**Gene**		**Sequence (5´→3´)**	**Amplicon (bp)**	**Tm (˚C)**	**Ref**
*16srRNA*	Forward	GCACAAGCGGTGGAGCATGTGG	299	55	[**14**]
	Reverse	CGTGTGTAGCCCTGGTCGTA			
*Serine protease*	Forward	CGATGCGCTTTCTCTCTCTC	110	62	
	Reverse	TCTCCACCTTGTCGATGTAGTA		62	**This work**
*Metlloprotease*	Forward	CAACCGCTTCACCCTCTATAC	119	62	
	Reverse	CCTGGCTTTCGTTCCACTT		62	

Bacterial Strain

The standard strain of A. hydrophila (ATCC 7966, RefSeq: GCF_000014805.1, GenBank: GCA_000014805.1, BioProject: PRJNA16697) was received from the Iranian National Center for Genetic and Biologic Resources (www.ibrc.ir). Molecular identification of the strain was performed by amplification and sequencing of the 16s rRNA gene using gene-specific primers (Table-1).

Antibacterial Potential and Determination of MIC

To screen the antibacterial activity of curcumin, a spreading culture of A. hydrophila was prepared on Muller Hinton medium, and then, wells (6mm) were prepared and poured with different concentrations of curcumin. After overnight incubation at 28°C, the growth inhibitory halos around the wells were measured. The agar macro-dilution method was employed to measure the minimum inhibitory concentration (MIC) of curcumin [13][14]. To prepare the curcumin stock solution, curcumin powder (51.2 mg) was dissolved in 5 ml of Dimethyl sulfoxide (DMSO) to obtain the stock solution of 10240 µg/ml [14]. During the preparation of the Muller-Hinton Agar medium, different concentrations of curcumin stock solution were added to obtain final concentrations of 128_1024 μg/ml. After streaking bacterial cells, the plates were incubated for 24 h and the MIC was determined as the minimum growth inhibitory concentration of curcumin.

Investigating the Antimicrobial Effect of Curcumin on A. Hydrophila

To characterize the antimicrobial potential of curcumin on A. hydrophila, a well diffusion test was performed. The spread culture of A. hydrophila was prepared on Muller Hinton agar medium. Wells with about 6 mm in diameter were prepared, and then 50μL of curcumin solution with concentrations of 1024, 512, 256, and 128 μg/mL were added to the wells. After incubation period the plates were monitored for bacterial growth inhibition zone.

Detection of Metalloprotease and Serine Protease Transcript Levels

A. hydrophila strains were grown in LB broth that contained curcumin (512 μg/ml) and then, bacterial cells were collected and their total RNA content was extracted using TriZol™ reagent (Thermo Fisher Scientific, USA), and treated with DNase I (Thermo Fisher Scientific, USA).

In order to measure the quality and determine the extracted concentration of RNA, a Nanodrop device was used. After ensuring the quality of the extracted RNA, synthesis of cDNA was done using RevertAid First Strand cDNA Synthesis Kit (Thermo Fisher Scientific, USA) as follows: 4 μL reaction buffer5 ×, 0.2 μg/μL Random Hexamer Primers, 2 μL dNTP mixture 1 mM, 1 μL RiboLock RTRNase inhibitor and 1 μL reverse transcriptase, with 12 μL nuclease-free water. Micro-tubes were incubated at 65 °C for 5 min, 25 °C for 10 min, 42 °C for 60 min, and the final elongation at 70 °C for 5 min [14]. The assay was performed in three replicates and the 16s rRNA gene was considered as house-keeping gene. The qPCR was performed using the following thermal cycling conditions: 50 °C for 120 s, 95 °C for 10 min, then followed by 45 cycles of denaturation at 95 °C for 20 s, annealing at 56 °C for 30 s, and extension at 72 °C for 40 s, also reaction mixture counting 10 μL of 2X SYBR Green qPCR master mix (Fermentas Co., Germany), 7 μL of nuclease-free water, 1 μL of each forward and reverse primer, and 1 μL of cDNA was used for each reaction. The sequence of the primers was displayed in Table-1. Finally, 2−ΔΔCT method was employed to calculate the relative expression of the genes [15].

Proteolytic Activities

The proteolytic activity of A. hydrophila in the presence of curcumin was evaluated by Skim milk agar plate assay. After the preparation of skim milk agar, wells with approximately 6 mm diameter were prepared. Then, 50 μL of supernatants from curcumin-treated and control cultures was added. The proteolytic halos around the wells were evaluated after incubation for 24h [16].

Statistical Analyses

Data are presented as mean ± SD after assaying in triplicates by using SPSS version 16 SPSS version 16 (IBM Corporation, United States). One-way analysis of variance (One-way ANOVA) was used to show the difference between curcumin-treated and control groups. P<0.05 were considered as statistically significant [17].

## Results

**Figure-1:**
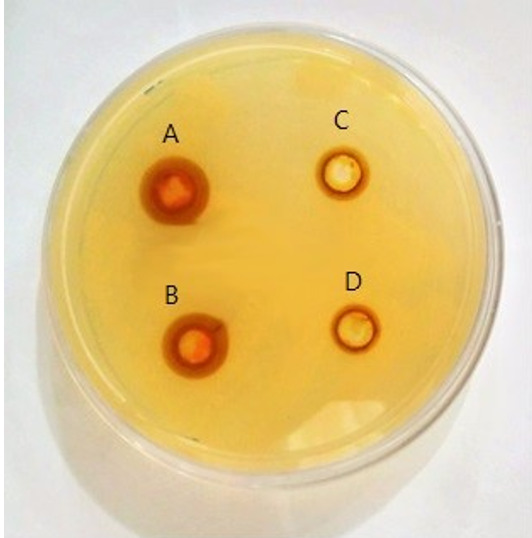
Figure 1. Investigating the antimicrobial effect of curcumin on A. hydrophila bacteria; Curcumin concentration in wells, A: 1024, B: 512, C: 256, D: 128

Antibacterial Potential

**Figure-2:**
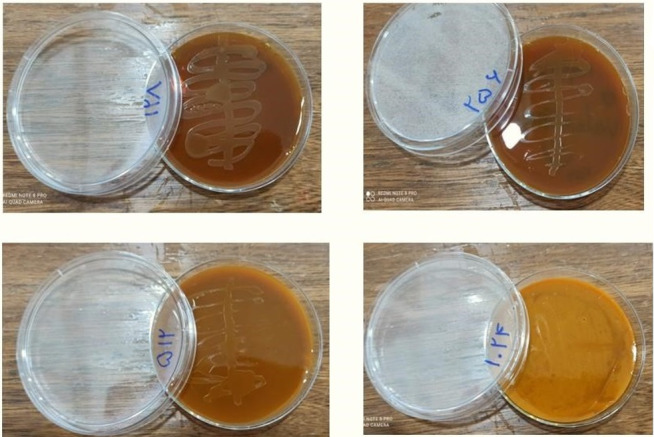
Figure 2. Results of Agar macrodilution assay

Investigating of the antimicrobial effect of curcumin on A. hydrophila showed that the maximum diameter of the growth inhibitory zone was related to the concentration of 1024 μg/ml of curcumin and the minimum one was associated with the concentration of 128 μg/ml (Figure-1). Also, the agar macro-dilution method performed on the A. hydrophila ATCC 7966 strain showed that curcumin at concentrations ≥1024 μg/ml, significantly inhibited bacterial growth (Figure-2). Therefore, a sub-inhibitory concentration of curcumin (512 μg/mL) was used for subsequent experiments.

Gene Expression

**Figure-3:**
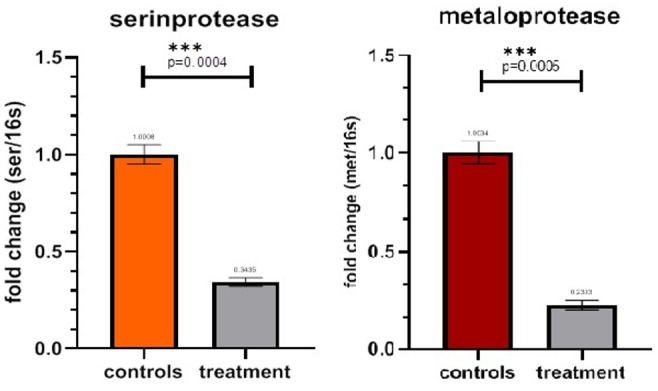
Figure 3. Gene expression analysis of metalloprotease and serine protease genes in A. hydrophila.

The relative expression of the metalloprotease and serine protease genes in the curcumin-treated and control groups was measured. Results indicated that the expression of both genes was remarkably reduced in curcumin-treated A. hydrophila 7966 (P<0.05). Treating A. hydrophila with curcumin remarkably reduced the expression of the metalloprotease and serine protease genes in A. hydrophila ATCC 7966 up to 66±0/01% and 77±0/01, respectively, compared to the control (Figure-3).

Bacterial Proteolytic Activity

**Figure-4:**
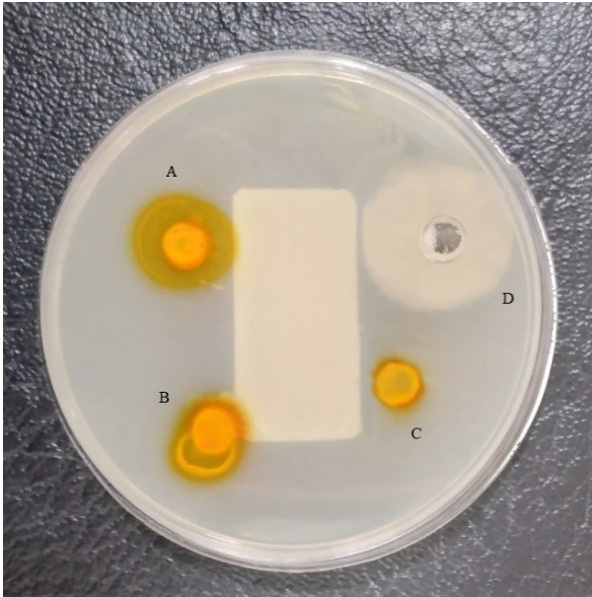
Figure 4. Proteolytic activity of A. hydrophila bacteria in Skim milk agar culture medium Curcumin concentration in the wells: A: 128, B: 256,C: 512 and D: 0 (control)

The results showed that, the proteolytic potential of A. hydrophila treated with different concentrations of curcumin (128-512 μg/mL) was considerably decreased by curcumin. Figure-4 displays the effect of curcumin on the proteolytic activity of A. hydrophila.

## Discussion

Considering the increased antibiotic resistance among bacterial pathogens, finding new alternative antimicrobial agents such as QS-inhibitor compounds (Quorum quencher) has been developed. Because of the easy production technology and leaving few side effects, natural compounds are always considered in medical fields and controlling biological infections. A large number of studies evaluated the antibacterial effect of curcumin [6][14], however, in this study, we characterized the effect of curcumin on the expression of the two major virulence factors of A. hydrophila, including serine protease and metalloprotease.

We found that curcumin has a considerable inhibitory effect on A.hydrophila ATCC 7966 with a MIC value of 1024 μg/mL. Exoproteases are significant virulence factors in A. hydrophila [5]. A previous study showed that there is a considerable association between the presence of aerolysin, hemolysin, and proteases, and the pathogenicity of A. hydrophila spp and if they are missing, the strains could lose their pathogenicity [18]. Serine protease and metalloprotease are major determinant virulent factors in A. hydrophila, which are encoded by the AHA_3857 and AHA_0978 gene loci, respectively. These two factors contribute to attacking the host, providing nutrients for bacteria through the growth phase, overcoming the host’s immune system, and causing infection. serine protease can hydrolyze casein and metalloprotease can attack elastin and casein of the host cell membrane [19].

Moreover, serine protease is associated with vascular leakage and reduced blood pressure by activating the kallikrein/kinin system which potentially comes up with septic shock [20]. Extracellular proteases significantly supply metabolic versatility which enables A. hydrophila to preserve in wide habitats, facilitate ecological interactions with other organisms, and have great adaptability to environmental changes. Proteases also protect the bacteria from the host’s immune system in the early stages of infection [5][7][8][21]. In A. hydrophila, the main QS system is an AHL-dependent system consisting of a signal synthase gene, ahyI, which synthesizes AHL molecules and a membrane regulator, which is encoded by the ahyR gene. Three types of AHL molecules are produced by the ahyI gene in A. hydrophila, the most important of them is N-butanoyl homoserine lactone (C4-HSL) [14]. Curcumin significantly inhibited QS related genes, ahyI, and ahyR, biofilm formation and reduction of proteolytic activity in A. hydrophila [14]. Swift et al. showed that creating mutation in the ahyR gene caused the lack of virulence factors production, like hemolysin, amylase, and proteases, also production of exoproteases can be blocked by C4-HSL analogs [22]. A similar study, that studied the effect of glucose on QS in A. hydrophila, indicated that glucose at a concentration of 0.25%(wt/vol) inhibited protease production and biofilm formation via the signal cascade QS inhibiting pathway which could be restored by adding C4-HSL [23]. This work revealed that curcumin can remarkably reduce the expression of the metalloprotease and serine protease genes in A. hydrophila. Curcumin has a considerable affinity for binding to AhyI protein [24] which may lead to reduced formation of the AhyR-AHL complex that may result in transcription attenuation of the bacterial QS system. The AHL-AhyR complex is the regulator of the transcriptional responses of QS _regulated genes [14]. Reduction of the AhyI protein and inefficiency of the AhyR-AHL complex could lead to transcription inhibition of QS_regulated genes and as results, transcription of Serine protease and metalloprotease decreased.

Recently, there has been growing interest in developing strategies for inhibiting bacterial QS to control bacterial infection. Due to the roles of serine protease and metalloprotease in the pathogenesis of P. aeruginosa, the reduction of the protease activity of A. hydrophila, which was observed in our study, probably leads to reduce the pathogenicity of this bacterium. The regulation of the activity of A.hydrophila exoprotease is important because its early elaboration leads to alteration of host defenses that may lead to inhibition of the bacterial infection [14][25]. Due to the role of bacterial proteases in tissue invasion and infection development, the significant decrease in the proteolytic activity of A. hydrophila, indicates the antivirulence effect of curcumin which can be used against bacterial pathogenesis.

## Conclusion

The expression of serine protease and metalloprotease genes in A. hydrophila, as two major virulence genes, was significantly reduced by curcumin and this led to a decrease in the bacterial proteolytic phenotype. Due to the antimicrobial and anti-QS properties of curcumin, this substance could be a promising anti-QS agent to control pathogenic microorganisms.

## Acknowledgment

The authors would like to thank the University of Guilan for letting us conduct this project.

## Conflict of Interest

The authors have declared that no competing
